# Environmental Engineering Approaches toward Sustainable Management of Spider Mites

**DOI:** 10.3390/insects3041126

**Published:** 2012-10-26

**Authors:** Takeshi Suzuki

**Affiliations:** 1 Japan Society for the Promotion of Science, Ichiban-cho 8, Chiyoda, Tokyo 102-8472, Japan; E-Mail: suzuki@restaff.chiba-u.jp; Tel.: +81-4-7137-8000; Fax: +81-4-7137-8008; 2 Center for Environment, Health and Field Sciences, Chiba University, Kashiwa-no-ha 6-2-1, Kashiwa, Chiba 277-0882, Japan

**Keywords:** diapause, IPM, natural enemy, photoperiod, ultraviolet radiation, water vapor

## Abstract

Integrated pest management (IPM), which combines physical, biological, and chemical control measures to complementary effect, is one of the most important approaches to environmentally friendly sustainable agriculture. To expand IPM, we need to develop new pest control measures, reinforce existing measures, and investigate interactions between measures. Continued progress in the development of environmental control technologies and consequent price drops have facilitated their integration into plant production and pest control. Here I describe environmental control technologies for the IPM of spider mites through: (1) the disturbance of photoperiod-dependent diapause by artificial light, which may lead to death in seasonal environments; (2) the use of ultraviolet radiation to kill or repel mites; and (3) the use of water vapor control for the long-term cold storage of commercially available natural enemies. Such environmental control technologies have great potential for the efficient control of spider mites through direct physical effects and indirect effects via natural enemies.

## 1. Introduction

Increased consumer demand for food security and safety has promoted the sustainable management of agricultural pests with reduced pesticide use. Traditionally, agricultural pest management has relied heavily on chemical control, which is convenient and has immediate effects. However, the frequent use of chemicals induces the development of pesticide resistance, eliminates beneficial organisms, promotes pest resurgence, and leads to the persistence of chemical residues in the environment.

Phytophagous mites infest virtually all crops, yet they were regarded as having secondary importance as orchard pests before the 1940s [1]. They became more serious with the advent of modern broad-spectrum pesticides and agricultural practices, and for the past few decades have been rated among the most serious crop pests [2]. Dichlorodiphenyltrichloroethane (DDT) and other chlorinated hydrocarbon insecticides are essentially ineffective against them, so their use for insect pest management raised mites to serious pest status [1]. Such outbreaks of once secondary pests apparently result from a complex of causes: not only direct effects of pesticides on natural enemies, pest fecundity, and host plant physiology, but also altered agronomic practices, have probably contributed to this situation [3,4,5,6].

In this situation, strategies for effective integrated pest management (IPM)—an approach that combines existing pest control measures, including timely application of small amounts of chemicals—are urgently needed. The Food and Agriculture Organization of the United Nations [7] defines IPM as “the careful integration of a number of available pest control techniques that discourage the development of pest populations and keep pesticides and other interventions to levels that are economically justified and safe for human health and the environment.”

In IPM programs, pest control measures are broadly divided into physical, biological, and chemical approaches [8]. Although physical control has been little exploited, the manipulation of lighting can provide effective control. Artificial lighting can influence pest and beneficial arthropods living in the crop indirectly via light-mediated changes in the plants [9] and directly by affecting the visual ecology and biological clocks in the arthropods [10]. Data on the latter are abundant. For example, nighttime lighting in orchards deters codling moths in egg laying [11] and repels noctuid moths [12,13]. Recent developments in light-emitting diode (LED) technology have drawn intense interest in its potential for the control of arthropod pests. LED technology has already been put to practical use in plant production systems for its capacity to produce monochromatic light of various wavelengths and to irradiate plants closely and uniformly [14,15]. The development, reproduction, diapause, behavior, and morphology of arthropods are often regulated by the light environment through photoperiod, light quality (*i.e.*, wavelength), and light intensity [16]. LEDs should therefore find a role in controlling harmful arthropods. Although the cost per unit light output of LEDs is still higher than that of some other light sources, it is still decreasing and LEDs offer benefits in pest control by virtue of their spectral characteristics, flexible shape, and low emittance of thermal infrared radiation. Indeed, LEDs have been often used for trapping insect pests [17,18,19,20,21,22,23,24,25,26]. Section 2 of this paper describes the potential applications of artificial lighting by LEDs in the control of spider mites (Acari: Tetranychidae) through the disturbance of their life cycles by photoperiod manipulation. Section 3 describes the use of ultraviolet (UV) radiation to control spider mites.

Biological control has been long practiced. In the past century, many exotic natural enemies of arthropod pests have been imported, mass reared, and released into agricultural fields and greenhouses as biological control agents [27,28]. Such actions were often highly successful in pest control, but sometimes had nontarget environmental effects (e.g., ecosystem disturbance) [27,28]. Therefore, the commercial use of indigenous natural enemies, which are already adapted to the domestic environment and can reduce such risks, has recently expanded [29]. Predatory mites (Acari: Phytoseiidae) are recognized as effective in the control of spider mites [30]. Commercialized mites (e.g., *Neoseiulus californicus*, *Phytoseiulus persimilis*) are effective [31], yet no more than 2 days' storage after receipt is recommended, as their survival is limited. This limitation often precludes farmers' scheduled application of predatory mites in fields and greenhouses. Section 4 of this paper describes the potential for water vapor control in the long-term cold storage of predatory mites.

## 2. Photoperiod Control

Organisms measure photoperiod to track the time of day and the passage of the seasons [16]. A great many organisms use photoperiodism as a calendar for their life cycles. In insects and mites, it determines the timing of diapause and the appearance of particular morphs. Diapause, which allows insects and mites to cope with unfavorable seasons, is defined as a hormonally mediated metabolic arrest with an increase in resistance to environmental stresses, a change in behavior, and suppression of morphogenesis [32]. It is so critical for the survival of insects and mites that any disturbance in its timing or expression could be harmful. Accordingly, diapause could be exploited in IPM [33]. If diapause is terminated too soon or its induction is prevented, this disruption could lead to ecological suicide in seasonal environments.

Barker *et al.* [34] proposed that disturbing diapause in pest species by artificially controlling photoperiod could offer a novel nonchemical control strategy. Lighting could be used to prevent diapause induction by interrupting the night or by extending the daylength [16]. Many studies have shown that photoperiodic manipulation can prevent diapause in the tortricid moth *Adoxophyes orana* [35,36], European corn borer *Ostrinia nubilalis* [37,38,39,40], codling moth *Cydia* (*Laspeyresia*) *pomonella* [37,40,41,42], tobacco budworm *Heliothis virescens* [41], pink bollworm *Pectinophora gossypiella* [41,43], oak silk moth *Antheraea pernyi* [41], rock pool mosquito *Aedes atropalpus* [44], and turnip sawfly *Athalia rosae* [45]. The artificial extension of daylength through the use of fluorescent tubes or mercury vapor lamps has prevented diapause of *O. nubilalis* and *C. pomonella* in the field [36,39], but such measures have not been developed further, probably because achieving sufficient light intensity in the field is too costly. LEDs may solve this problem, because their energy consumption is low and their life span is long; high light intensity can be obtained, because plants can be irradiated closely; wavelengths can be matched to the spectral sensitivity of pests; and light sources of various sizes and shapes can be fabricated [46].

Adult female spider mites enter reproductive diapause [47] and overwinter without feeding, and they do not oviposit if they have been exposed to long-night photoperiods as juveniles. Immature females of the two-spotted spider mite *Tetranychus urticae* and the Kanzawa spider mite *T. kanzawai* exposed to long-day photoperiods do not enter diapause as adults, and they continue to feed and start ovipositing [48,49]. Night-interrupting light often induces short-night (long-day) effects in these species, thus preventing diapause induction at the adult stage even under long-night photoperiods [50,51,52]. If the induction of diapause is prevented before winter arrives, adult females cannot overwinter. Diapause termination of *T. urticae* is also regulated by photoperiod [53,54]. If diapause is terminated during winter, it can be difficult for adult females to overwinter. These effects could be used to stifle population growth during the following spring. Therefore, disrupting diapause induction or termination by artificial light is a potential technique for the nonchemical control of spider mites.

**Figure 1:**
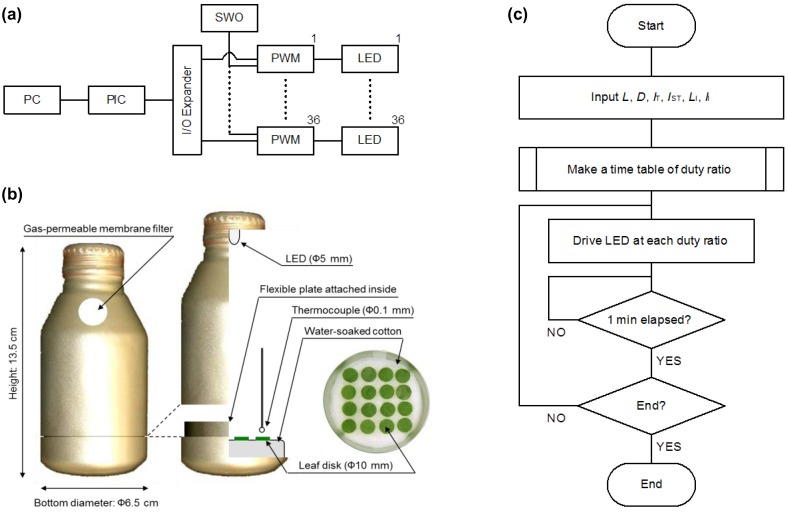
Space-saving photoperiodic bottle system. **(a)** Schematic diagram of system. PC, netbook computer; PIC, peripheral interface controller; SWO, saw-wave oscillator; PWM, pulse-width modulator (×36); LED, light-emitting diode (×36). **(b)** Side view and sectional view of bottle, with removable bottom for setting samples. **(c)** Algorithm of control software. *L*, light period; *D*, dark period; *I*_T_, night-interrupting light period; *I*_ST_, period between beginning of *D* and beginning of *I*_T_; *L*_I_, duty ratio during *L*; *I*_I_, duty ratio during *I*_T_. (Modified from [46,52].)

To identify the most effective period, intensity, timing, and wavelength of additional lighting at disrupting diapause, extensive phenomenological investigations with laboratory experiments are needed. To date, such experiments have relied on large-scale equipment with a large light source (e.g., fluorescent tubes), timing device, and incubator for each photoperiodic condition examined, just to observe tiny organisms such as mites. Such large systems are costly and bulky. In addition, it is difficult to create specific wavelengths with fluorescent lamps. Such large systems are wasteful and are not suitable for precisely observing the photoperiodic responses of small organisms. To solve these problems, my colleagues and I used the advantages of LED technology to develop a space-saving photoperiodic bottle system (Figure 1) [52].

**Figure 2:**
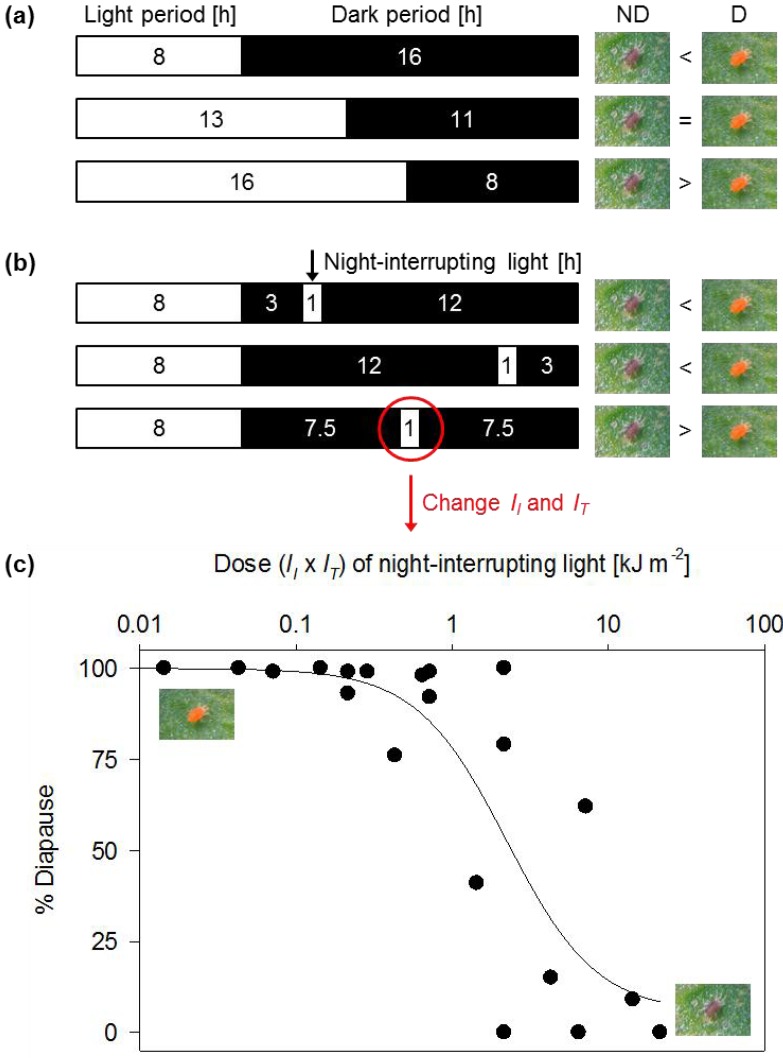
Effects of **(a)** extending the light period, **(b)** the timing of a 1-h night-interrupting light, and **(c)** the dose of night-interrupting light applied in the middle of the night on the incidence of diapause in a Japanese population of the Kanzawa spider mite *Tetranychus kanzawai*. ND, nondiapausing adult females (dark brown); D, diapausing adult females (brilliant orange). Dose of night-interrupting light was calculated by multiplying the intensity (*I*_I_) by the period (*I*_T_). (Modified from [49,51].)

The system was intentionally designed to use widely available, inexpensive microcontrollers (e.g., PIC) and other equipment (e.g., LEDs, aluminum bottles), significantly reducing the total cost. We wrote the software using a free tool distributed on the Internet. Despite the small investment, the system can quickly create various photoperiodic conditions in each bottle. As all bottles are held in a single incubator, and as a single LED that generates little radiant heat is used in each bottle, uniform air temperatures with little fluctuation (<0.1 °C) are maintained among bottles and between light and dark periods [52]. This design does away with different heat conditions in each incubator and unwanted radiant heat from lamps during the light period. This is a crucial improvement, because the induction of diapause in some insects and mites is influenced not only by photoperiod, but also by periodic fluctuations in air temperature, specifically thermoperiodism [55,56,57]. When the phytoseiid mite *Amblyseius potentillae* is reared in continuous darkness, the incidence of diapause in conditions with a difference of 4 °C to 12 °C between thermophase (8 h d^−1^) and cryophase (16 h d^−1^) is higher than that under constant air temperature [57]. Although the effects of thermoperiodic conditions with slight temperature fluctuations (<4 °C) between thermophase and cryophase have not been reported, an accurate investigation of photoperiodic effects on diapause induction requires the removal of air temperature fluctuations. The photoperiodic bottle system achieves this requirement. Using this system, we investigated the effects of extending the light period (*i.e.*, daylength), timing of a 1-h night-interrupting light, and dose of night-interrupting light on diapause in *T. kanzawai* (Figure 2) [49,51].

The results suggest that the critical dark period (CDP; the period that induces a 50% incidence of diapause), the night-interrupting timing that makes each sequential dark period shorter than the CDP, and the dose of night-interrupting light are all important factors in disrupting diapause induction in spider mites. The CDP shows geographic variation; it tends to be shorter as the latitude increases, in *T. kanzawai* [49,58,59] and *T. urticae* [48,58,60,61,62,63]. The incidence of diapause in *T. urticae* also varies with air temperature [63], light intensity, and wavelength [64]. The air temperature effect seems to be indirect, acting through regulation of the developmental time of stages that are sensitive to photoperiod, rather than direct, because the number of long-night photoperiods accumulated by the photoperiodic counter [65] for the induction of diapause varies with the duration of sensitive stages. The effective timing of the night-interrupting light (Figure 2b) varies with photoperiod [50]. In addition, the effect of the night-interrupting light on insects varies with wavelength [66,67]. Before artificial lighting can be applied to agricultural fields, therefore, we need further investigation of the diapause responses to the different photoperiods with additional lighting under different air temperatures, light intensities and wavelengths, using spider mites in a field to find the most appropriate lighting condition.

## 3. UV Control

It has long been known that ants respond behaviorally to UV radiation [68]. Most insects can see UV radiation and respond to it [69,70]. Therefore, UV radiation could be used to disturb the behavior of pest insects. For example, whiteflies, thrips, and aphids show a distinct preference for UV radiation [71,72]. Lack of UV radiation alters the normal behavior of whiteflies, reducing flight [73].

On the other hand, UV radiation, particularly UV-B radiation (280–315 nm), is harmful to organisms. UV-B radiation directly damages DNA and is absorbed by certain coenzymes and pigments *in vivo*, raising these molecules to an excited state; the excitation energy is eventually transferred to water molecules, yielding reactive oxygen species (ROS). The UV-mediated formation of ROS also causes DNA damage [74], which can be lethal, particularly to small organisms such as mites. Therefore, UV radiation may have pesticidal effects.

**Figure 3:**
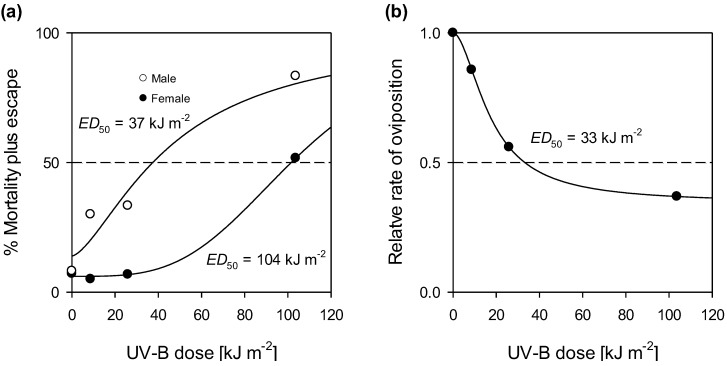
Effects of 3-day UV-B irradiation (4 h d^−1^) on **(a)** mortality plus escape rate and **(b)** oviposition by adult two-spotted spider mite (*Tetranychus urticae*). Oviposition rates are shown relative to the 3-d rate (18.9 ± 0.4 eggs per female) under continuous darkness (UV-B dose = 0 kJ/m^2^). *ED*_50_ = median effective dose. (Modified from [77]).

The susceptibility of *T. urticae* to UV-B has been well investigated [75,76,77,78,79]. We exposed *T. urticae* to UV-B radiation (λ_max_ = 300 nm) [77] using the Okazaki Large Spectrograph [80]. Mortality and escape of both males and females were induced (Figure 3a) and oviposition was inhibited (Figure 3b) as the dose increased. However, UV-A radiation (λ_max_ = 350 nm) did not affect mortality or oviposition [77]. Under UV-B irradiation, the *ED*_50_ values (median effective dose) for mortality plus escape were 37 kJ/m^2^ in males and 104 kJ/m^2^ in females. The *ED*_50_ value for oviposition was 33 kJ/m^2^. These *ED*_50_ values are comparable to the doses of UV-B radiation observed in the field over 2 to 5 days in summer, when spider mites shelter beneath leaves [77]. This behavior suggests that spider mites avoid UV-B radiation, which could prove an effective tool for mite control. Although no deleterious effects of UV-A radiation were observed, it repels adult females [77,78]. Therefore, both UV-B and UV-A radiation could be used to repel spider mites.

## 4. Water Vapor Control

Methods for the mass rearing of natural enemies are important for biological control [81,82,83,84], and abundant data on the optimum conditions for their development and reproduction have been gathered. Despite the successful rearing of natural enemies, there is still great potential for reducing the costs and increasing their availability, through efficient storage, for example [85]. It could also allow flexibility in the release time to synchronize release with optimum weather conditions, cultivation status, and pest outbreaks [86,87,88,89,90]. Cold storage can extend the life span of natural enemies and is widely recommended for cost-effective biological control [85,88,89,90,91,92,93]. However, the role of other environmental factors such as water vapor condition (*i.e.*, humidity) is not well understood [94]. Preventing the loss of body water to the atmosphere may pose a challenge to long-term cold storage [94,95].

Research on the humidity-controlled cold storage of phytoseiid species identified higher relative humidity (RH), which equates to lower vapor pressure deficit (VPD), as important for storing *P. persimilis*, *N. californicus*, and *Amblyseius cucumeris* (Table 1). With feeding, 93% of adult female *P. persimilis* fed on *T. kanzawai* eggs during storage at 10°C and 0.0 kPa VPD for 70 days survived [96]; and even without feeding, 60% survived under the same conditions for 50 days [96]. In addition, 50% of adult female *N. californicus* survived 65 days without food at 5 °C and 0.0 kPa VPD [92]. High-humidity cold storage did not compromise the qualities of stored *N. californicus* females or their progeny [93]. A lower VPD probably suppresses evaporative water loss from mites and maintains their body water homeostasis, leading to a longer life span with less desiccation damage. Storage without food is also considered to avoid the potential risk of accidental shipment of harmful prey species (such as spider mites).

**Table 1:** Humidity-controlled cold storage of phytoseiid mites

Species	Stage (♀/♂)	Air temperature (°C)	RH (%)	VPD (kPa)	Food or chemical	Storage (d)	Survival (%)	Ref.
*P. persimilis*	Adult (♀)	10	100	0.0	*T. kanzawai* eggs	70	93	[96]
*P. persimilis*	Adult (♀)	7.5	100 *	0.0 *	*T. urticae* eggs	56	43	[91]
*P. persimilis*	Adult (♀)	10	100	0.0	–	50	60	[96]
*P. persimilis*	Adult (♀)	8	70	0.3	–	35	51	[97]
*P. persimilis*	Adult (♀)	8	70–90	0.1–0.3	Cryoprotectant	29	46	[98]
*P. persimilis*	Adult (♀)	8	100	0.0	–	14	80	[99]
*P. persimilis*	Egg	10	100	0.0	–	25	98	[96]
*N. californicus*	Adult (♀)	5	100	0.0	–	75	35	[93]
*N. californicus*	Adult (♀)	5	100	0.0	–	65	50	[92]
*N. californicus*	Adult (♀)	10	92	0.1	–	30	83	[85]
*N. californicus*	Adult (♂)	5	100	0.0	–	32	50	[100]
*A. cucumeris*		9	100*	0.0*	–	70	63	[101]
								

* Not stated in the reference, but inferred from the setup.

Higher RH is suitable for long-term cold storage of phytoseiid mites (Table 1). To control RH simply, saturated salt solutions have long been used, with the composition corresponding to a specific RH at a specific air temperature. However, this method usually needs a long time to reach the target RH, it provides only a static RH in a small container, and salt solutions can have unwanted effects on organisms.

**Figure 4:**
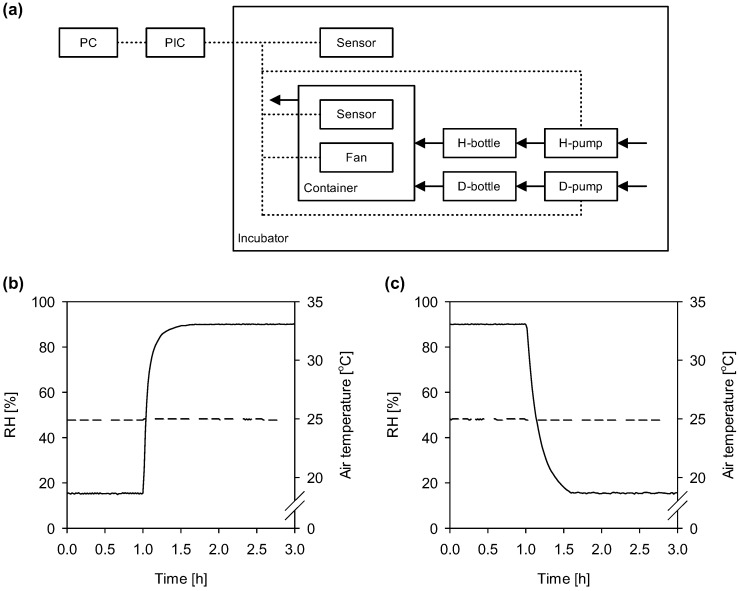
**(a) **A computer-based system for controlling humidity by combining streams of humidified and dehumidified air in an acrylic container. PC, netbook computer; PIC, peripheral interface controller; Sensor, relative humidity (RH) and air temperature sensor; H-bottle, bottle filled with water for humidifying air; D-bottle, bottle filled with silica gel for dehumidifying air; H-pump, pump for blowing air into the H-bottle; D-pump, pump for blowing air into the D-bottle. Broken lines represent electrical signals. Arrows indicate air flow. **(b**, **c)** Performance of humidification and dehumidification: time courses of RH (solid line) and air temperature (broken line) inside the container when air was **(b)** humidified from RH set-point of 15% to 90% or **(c)** dehumidified from RH set-point of 90% to 15% at 25 °C. Humidification and dehumidification were started 1 h after recording began. (Modified from [102].)

To resolve these problems, we developed a computer-based system to control RH by combining streams of humidified and dehumidified air in a container (Figure 4a) [102]. In this system, humidification from RH of 15% to 90% (Figure 4b) and dehumidification from RH of 90% to 15% (Figure 4c) at an air temperature of 25 °C were properly operated with short time constants of 4.3 and 10 min, respectively. For the system, we also developed the software which has a function that can create a time table of the RH set point every 10 min. Although the time scheduling function with the quick responses in humidification and dehumidification may not be needed in storage of natural enemies, these will be useful for investigating organisms' responses to periodic changes in water vapor conditions, such as responses to photoperiods and thermoperiods (*i.e.*, photoperiodism, thermoperiodism). We also anticipate that our system will be a powerful tool for the large-scale storage of natural enemies to balance supply and demand.

## 5. Conclusions

The light environment tells spider mites when to develop, reproduce, and enter diapause, but carries the risk of lethal UV-B radiation. Therefore, manipulation of it offers a potentially effective means of controlling pest mites. Investigation of effective irradiation patterns, effects of artificial lighting for pest mite control on natural enemies and plants, and electricity costs would support the design of effective illumination for IPM.

The control of water vapor conditions offers great potential for the long-term cold storage of phytoseiid mites. Maintenance of a low VPD (<0.1 kPa) and a low air temperature (5–10 °C) suppresses water loss and the use of energy reserves by phytoseiid mites, prolonging their life span without feeding during storage, without any decrease in the quality of the mites. Recently, we developed a handy vessel that maintains a low VPD without wetting the contents, for the long-term storage of phytoseiid mites (Japanese Patent Application No. 2012-102840).

Rosette-forming herbaceous plants offer effective overwintering sites for *N. californicus *in orchards probably because the underside of leaves provides suitable climatic conditions by decreasing VPD and protecting from UV-B radiation [103]. Interestingly, nondiapausing adult female of *N. californicus* survived winter better under fallen leaves or artificial shelters on the ground, where the VPD is lower [104] and UV-B radiation may be filtered out. The lower VPD and lower dose of UV-B radiation are probably critical to overwintering by phytoseiid mites in the field, as shown in laboratory experiments [76,85,92,93]. Therefore, the provision of such artificial hibernacula may be useful for conserving phytoseiid mite populations during winter.

Limiting the elevation of an organism to pest status is the most sustainable and profitable pest management strategy, benefiting all participants in the ecosystem, including humans [8]. This primary line of defense against pest outbreaks should be boosted by the development of farming practices and cropping systems based on a comprehensive understanding and shoring up of natural, “built-in” regulators that offer inherent plant defense, such as plant mixtures, soil, and natural enemies [8]. If the limitation is removed, comprehensive pest control measures (*i.e.*, IPM) should be prepared as backups. To further enhance the IPM of spider mites, a “glocal” system needs to be developed to globally share local information on pest spider mites, natural enemies, the compatibility of natural enemies with existing narrow-spectrum acaricides, other possible control measures, and the effects of each of these factors under different environmental conditions.
